# Driving self-restriction and age: a study of emergency department patients

**DOI:** 10.1186/s40621-014-0018-z

**Published:** 2014-09-02

**Authors:** Marian E Betz, Christopher R Carpenter, Emma Genco, David B Carr

**Affiliations:** 1 Department of Emergency Medicine, University of Colorado School of Medicine, 12401 E. 17th Ave B-215, Aurora, 80045 Colorado USA; 2 Division of Emergency Medicine, Washington University School of Medicine, Campus Box 8072, 660 S. Euclid Avenue, St. Louis, 63110 MO USA; 3 VISN 19 Mental Illness Research, Education and Clinical Center, Denver, 80220 CO USA; 4 Department of Medicine and Neurology, Division of Geriatrics and Nutritional Science, Washington University School of Medicine, 4488 Forest Park Ave, St. Louis, 63108 MO USA

**Keywords:** Driving, Self-restriction, Avoidance, Emergency department, Older driver, Emergency department

## Abstract

**Background:**

Driving self-restriction is well-documented among older drivers but might also occur among younger drivers. Little is known about the driving patterns of emergency department (ED) patients, who may be a high-risk population for motor vehicle crashes (MVCs). We sought to compare the driving patterns and MVCs of younger and older adult ED patients in order to inform development of injury prevention interventions in EDs.

**Methods:**

We surveyed English-speaking younger adult (age 25–64) and older adult (age ≥65) ED patients, excluding non-drivers and those who were cognitively-impaired or too sick to participate. We compared drivers by age group and used logistic regression with adjustment for driving frequency to examine factors associated with driving self-restriction.

**Results:**

Of those eligible, 82% (n = 178) of younger adult and 91% (n = 134) of older adult patients participated; approximately half were women. Similar proportions of younger and older adult patients reported driving everyday/almost everyday (80%) but also self-restricting driving in inclimate weather (48%), heavy traffic (27%), in unfamiliar places (21%), when travelling with passengers (1.6%) or when alone (1.3%). Fewer younger adult than older adult patients avoided driving at night (22% versus 49%) or on highways (6.7% versus 26%). In multivariable logistic regression, factors significantly associated self-imposed driving restriction in ≥1 driving situation were female gender (Odds Ratio [OR] 2.40; 95% CI 1.42-4.05) and ever feeling “confused, nervous or uncomfortable” while driving (OR 1.87; 95% CI 1.03-3.39). There was a non-significant trend for differences in proportions between younger adult (11%) and older adult (6.8%) drivers reporting ≥1 MVC as a driver in the past 12 months.

**Conclusions:**

Similar proportions of younger and older adult ED patients self-restrict driving, albeit in different situations, which has implications for behavioral interventions for injury prevention and for education of patients and medical providers.

**Electronic supplementary material:**

The online version of this article (doi:10.1186/s40621-014-0018-z) contains supplementary material, which is available to authorized users.

## Background

With the growing older adult population and the recognition that some older drivers are at elevated risk of motor vehicle crashes (MVCs), there has been increasing attention to the issue of older driver safety. Previous studies have demonstrated that many older drivers self-restrict their driving, perhaps as a way to compensate for recognized declines in safe driving ability (Betz and Lowenstein [2010]; Braitman and McCartt [2008]; Braitman and Williams [2011]; Charlton et al. [2003]; Charlton et al. [2006]; Donorfio et al. [2008a]; Forrest et al. [1997]; Lyman et al. [2001]; Naumann et al. [2011]). There has also been some discussion about the concept of graduated driving reduction (akin to graduated driver licensing in teenagers) as an injury prevention policy strategy for older drivers (Langford and Koppel [2011]). To date, although there is discussion of the influence of personality on driving self-restriction (Molnar et al. [2014]), most studies of this behavior have focused on older drivers.

In a previous small study examining driver screening in one emergency department (ED) setting, fewer younger drivers reported self-restriction in certain driving conditions as compared to older drivers but no additional analysis concerning the effect of age on self-restriction was conducted (Betz and Fisher [2009]). In a more recent national survey, younger and older drivers were compared in terms of their self-restriction in driving situations. Younger drivers were as likely as older drivers to avoid driving in bad weather, but less likely to avoid driving at night or on high speed roads (Naumann et al. [2011]). Other driver characteristics, including female gender and cognitive or visual deficits, have been associated with self-restricted driving by older adults in other studies (Braitman and McCartt [2008]; Braitman and Williams [2011]; Gwyther and Holland [2012]; Lyman et al. [2001]; Stutts [1998]; West et al. [2003]), but less is known about younger drivers who self-regulate their driving.

A few prior studies have documented some of the driving patterns and crash experiences of older adult drivers, including those evaluated in EDs (Betz and Fisher [2009]; Betz et al. [2012b]; Stiffler and Wilber [2011]; Vogel et al. [2013]). Older drivers have been noted to have fewer total MVCs but higher crash rates per mile driven and per licensed driver in comparison to younger drivers in general population studies (U.S. Census Bureau [2012]; Dellinger et al. [2004]). Among ED patients treated after an MVC, older adults are more likely than younger adults to be admitted to the hospital (Vogel et al. [2013]). Less is known about how driving patterns vary by age in ED patients, who may be a population at higher risk of MVCs and other adverse outcomes because of higher levels of comorbidities (Garcia et al. [2010]), acute injuries or illness (Garcia et al. [2010]), and social stressors (Institute of Medicine Committee on the Future of Emergency Care in the United States Health System [2007]).

The ED population may be an important target for interventions related to driver safety, and there have been renewed calls for ED providers to be involved in the assessment of older drivers (Lotfipour et al. [2014]). Indeed, in many cases, EDs may be an ideal site for screening and intervention for public health problems (Bernstein et al. [2007]; Bernstein and Haukoos [2008]; Gaddis and Hauswald [2008]; Pollock et al. [2001]). Lack of insurance, difficulty obtaining outpatient appointments and other barriers to care (Institute of Medicine Committee on the Future of Emergency Care in the United States Health System [2007]) mean that for many patients, an ED visit may be their only contact with a medical provider. Patients are often seen in EDs after falls or trauma; for example, in 2012, emergency departments treated 2.4 million nonfatal fall injuries among older adults ([WISQARS]). Such injuries may be a “red flag” for driving risk, as older adults who fall are at in increased risk for an MVC (Cross et al. [2009]). Drivers may also be seen in the ED for injuries that may adversely affect their driving safety. In addition, injuries or illnesses that trigger an ED visit may also represent “teachable moments” at which a patient is more open to behavioral change and ED-based interventions may be able to reduce future risk. At the same time, interventions need to be designed with attention to the behaviors and beliefs of the targeted population. Older drivers are open to guidance from physicians and to driving safety materials to take home from EDs (Stiffler and Wilber [2011]). Driving safety materials specific to the older ED population do not yet exist, but they could include recommendations about self-restriction.

We hypothesized, however, that younger and older ED patients may be similar in their self-restriction, suggesting this topic might not be a useful target for educational materials. We therefore sought to: (1) compare younger and older ED patients’ driving prevalence, patterns and experiences, including self-restricted driving and MVCs; and (2) identify driver characteristics associated with self-imposed driving restrictions. These results should provide novel information about the potentially high-risk population of ED patients, which could be useful in educational approaches through development of materials or programs for ED patients or through further education of medical providers and students (Lotfipour et al. [2014]).

## Methods

### Study design, setting and participants

This cross-sectional study examined patients evaluated in the ED (annual census of 87,000 visits) at a tertiary-care university hospital in Colorado. Eligible subjects were registered ED patients aged ≥25 years who reported driving a motor vehicle in the past 30 days. Patients who did not speak English, who had significant acute illness or trauma that interfered with their ability to consent or participate, or who had cognitive dysfunction (defined by a Six-Item Screener [Carpenter et al. [2011] score less than four) were excluded. Enrollment occurred during shifts (7 a.m. to 7 p.m.) distributed equally among the days of the week. Due to research staff limitations, enrollment did not occur daily, and enrollment days were distributed over several months. During these enrollment shifts, all eligible patients visiting the ED were invited to participate. Older (≥65 years) patients were enrolled between January, 2010 and October, 2011, as part of a larger study to develop a brief older driver screening tool (Betz et al. [2012a]). A comparison group of drivers (age 25–64 years) was enrolled from February to March, 2012. We refer to this group as “younger” relative to the older driver group, and we chose to exclude the youngest drivers (<25 years) because we hypothesized the driving patterns and experiences of novices would be significantly different. After informed consent, participants completed a short survey (Additional file 1) with questions about demographic, health-related and driving characteristics; participants could self-complete the survey or have it read aloud by research staff. Encounters took place in ED patient rooms away from other patients to protect subject privacy. Participation was voluntary, and all participants were assured that their responses would not be shared with non-study physicians, family members, ED or clinic staff or hospital or law enforcement authorities (including the Department of Motor Vehicles). This project was approved by the Institutional Review Board.

### Survey development and variable definitions

Some survey questions were obtained from existing questionnaires (Betz and Fisher [2009]; Behavioral Risk Factor Surveillance System BRFSS [2009]; Eby [2000]) and additional questions related to driving behaviors were created *de novo*. The instrument, including newly-created questions, was pilot-tested among 20 younger and older ED patients for clarity and content validity prior to actual implementation, with no recommendations for modification. Demographic characteristics that were documented included patient age and gender.

Participants were asked about their driving experiences, including their self-rated driving ability (“good,” “average,” or “poor”) and the presence and number of MVCs and police stops as a driver in the past 12 months. Participants were also asked “In the past 12 months, has anyone recommended you stop driving or give up your car keys?” Additionally, they were queried as to how often they feel “confused, nervous or uncomfortable” while driving and how often they have trouble “reading the license plate of the car in front while stopped”. Participants responded “often,” “sometimes,” “rarely” or “never.” For analytic purposes, responses to these two questions were dichotomized as “ever” (“often,” “sometimes” or “rarely”) versus “never”. For self-restriction, participants were asked if they “usually avoid driving” in each of the following situations (with the option to answer yes to more than one): at night; on high speed roads; in heavy traffic; in bad weather; with others; alone; or in unfamiliar places.

Our primary outcome was self-restricted driving in ≥1 situation. For logistic regression analyses, we separately included age as a continuous variable and as age groups (younger versus older). All data, including the outcome, were self-reported. Study participants were blinded to the purpose of the study, although they were told that we sought to understand the driving patterns and experiences of drivers.

### Data management and statistical analyses

Study data were managed using Research Electronic Data Capture, a secure, web-based application (Harris et al. [2009]), and exported to Stata for analyses. We described driver characteristics using proportions and 95% confidence intervals (CIs) for categorical variables, or medians and interquartile ranges (IQRs) for continuous variables. To estimate unadjusted associations between driver characteristics and the primary outcome (self-restricted driving in ≥1 situation), we performed bivariate analyses with chi square (or Fisher exact in cases of less than five observations in a cell) tests for statistical significance and calculated odds ratios (ORs) with 95% CIs to measure the strength of associations. We hypothesized that adverse driving experiences (including MVCs, police stops, or being asked to stop driving) might affect driving behavior so we examined these characteristics in our regressions. Next, we developed a multivariable logistic regression model to estimate independent associations between driver characteristics and self-restricted driving in ≥1 situation, with inclusion of all variables with a P-value of <0.2 in bivariate analysis and adjustment for driving frequency.

## Results

Of patients evaluated in the ED during enrollment sessions, 14% of younger patients and 66% of older patients were ineligible because they weren’t current drivers, didn’t speak English, had cognitive impairment, or were too sick to answer. In total, 82% (N = 178) of eligible younger patients and 91% (N = 133) of eligible older patients participated. The sample was approximately half female (58% among younger patients, 51% among older; Table 1). The median age was 44 years for younger patients (interquartile range [IQR] 21) and 72 for older patients (IQR: 9).

**Table 1:** **Characteristics, opinions and experiences of participants, by age group (n = 311)**

	Younger drivers		Older drivers	
	(25–64 years) n = 178		(≥65 years) n = 133	
	n	%	n	%
Age in years (median, IQR)	44	21	72	9
Female	103	58	68	51
Current frequency of driving				
Everyday or almost everyday	147	83	101	76
Occasionally	23	13	24	18
Seldom	8	4.5	8	6
Self-rated general driving ability**				
Good	158	89	129	97
Average	18	10	3	2.3
Poor	0	0.0	1	0.8
MVCs (as a driver) in past 12 months				
0	155	87	123	92
1	18	10	7	5.3
2	2	1.1	2	1.5
Police stops (as a driver) in past 12 months*				
0	132	74	117	88
1	38	21	13	10
2	4	2.3	1	0.8
3	2	1.1	1	0.8
In past 12 months, asked by someone to stop driving	2	1.1	4	3.0
Frequency of feeling confused, nervous or uncomfortable while driving				
Often	0	0.0	0	0.0
Sometimes	14	7.9	16	12
Rarely	46	26	29	22
Never	112	63	88	62
Frequency of having vision problems while driving**				
Often	2	1.1	2	1.5
Sometimes	9	5.1	4	3.0
Rarely	24	13	5	3.8
Never	137	77	122	92
Number of conditions where driving avoided**				
0	58	33	43	32
1	64	36	29	22
2	29	16	17	13
≥3	27	15	44	33

**Table** **1** **Legend:**

IQR: Interquartile Range; MVC: Motor Vehicle Crash.

Numbers may not add to 100% or total because of rounding or missing data (not displayed if <5% of total).

*P < 0.05, **P < 0.01 under Chi-square (or Fisher exact if <5 observations in a cell).

Similar proportions of younger (83%, 95% CI 77–88) and older (76%, 95% CI 69–83) patients reported driving everyday or almost everyday (Table 1), although among older patients fewer women (68%; 95% CI 56–79) than men (85%; 95% CI 76–94) reported this frequency level. The majority of all drivers reported they were “good” drivers, but this was endorsed by significantly fewer younger (89%, 95% CI 84–93) than older (97%, 95% CI 94–100). Although more younger (11%, 95% CI 6.7-16) than older (6.8%, 95% CI 2.5-11) drivers reported having ≥1 MVC as a driver in the past year, the difference was not statistically significant. For police stops, however, a significantly greater proportion of younger (25%, 95% CI 19–31) than older (11%, 95% CI 5.9-17) drivers reported ≥1 stop as a driver in the past 12 months. Among younger drivers, males were more likely than females to report ≥1 police stop as a driver in the past 12 months (31%, 95% CI 20–42; versus 21%, 95% CI 13–29); there were no other gender differences among age groups for MVCs or police stops.

Only 1.1% (n = 2; 95% CI 0.1-4.0) of younger and 3.0% (n = 4; 95% CI 0.8-7.5) of older participants said that in the past 12 months someone had asked them to stop driving, without differences by gender (Table 1). However, approximately one-third of younger (35%, 95% CI 28–42) and older (34%, 95% CI 26–42) patients reported ever feeling confused, nervous or uncomfortable while driving. While similar proportions of male and female older drivers reported this (p = 0.716), fewer young males (21%, 95% CI 11–31) than young females (45%, 95% CI 35–54) did. When asked about vision troubles while driving, more younger (20%, 95% CI 14–26) than older (8.3%, 95% CI 3.5-13) drivers reported ever having difficulty reading the license plate of the car in front while stopped, without significant differences by gender in either age group.

**Figure 1:**
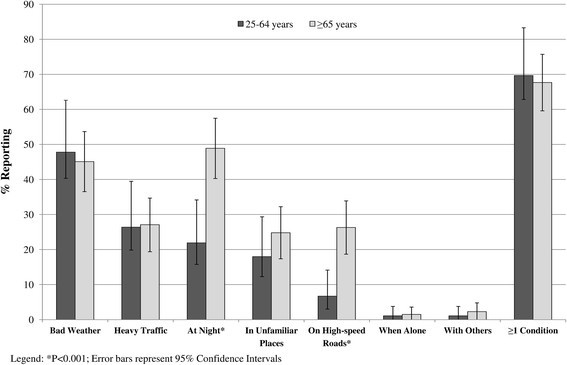
**Participants reporting “usually” self-restricting driving, by driving condition and age group (n = 311).**

Older drivers were more likely to avoid driving in a greater number of conditions (Table 1), but overall, 69% (95% CI 64–74) of all patients reported usually avoiding driving in at least one hazardous situation, without significant differences by age in unadjusted analysis (Figure 1). Similar proportions of younger and older drivers reported restricting their driving: in inclimate weather (47%; 95% CI 41–52); in heavy traffic (27%; 95% CI 22–32); in unfamiliar places (21%; 95% CI 16–25); when travelling with passengers (1.6%; 95% CI 0.2-3.0); or when alone (1.3%; 95% CI 0.0-2.5). However, fewer younger than older drivers restricted their driving at night (22%, 95% CI 16–28; versus 49%, 95% CI 40–57) or on highways (6.7%, 95% CI 3.0-10; versus 26%, 95% CI 19–34).

In unadjusted logistic regression, age in years and age group were not associated with self-restricted driving in at least one condition (Table 2). However, all women (regardless of age) were more likely to report self-restricting driving in at least one situation (OR 2.72, 95% CI 1.66-4.45), as were those who drove only occasionally (OR 3.08, 95% CI 1.33-7.18) or seldom (OR 2.34, 95% CI 0.65-8.44) compared to those who drove “everyday or almost everyday.” Drivers who reported ever feeling “confused, nervous or uncomfortable” while driving (OR 2.22, 95% CI 1.28-3.84) or ever having vision trouble while driving (OR 2.92, 95% CI 1.25-6.79) were also more likely to report self-restricting their driving. Having ≥1 MVC or police stop as a driver in the past 12 months or being asked by someone to stop driving were not significantly associated with self-restricted driving. In multivariable logistic regression, after adjusting for driving frequency, factors significantly associated with self-restriction of driving were female gender (OR 2.40; 95% CI 1.42-4.05) and ever feeling confused, nervous or uncomfortable while driving (OR 1.87; 95% CI 1.03-3.39).

**Table 2:** **Driver characteristics associated with usually self-restricting driving in ≥1 situation† (n = 311)**

Demographic or driving-related characteristic	No self-restriction (n = 97)	Self-restriction in ≥1 situation (n = 214)	Unadjusted odds ratio (95% CI)	P value	Adjusted odds ratio
	n (%)	n (%)			(95% CI)
Age in years (median [SD])*	61 (25)	59 (30)	1.00 (0.98-1.01)	0.659	
Age Group					
25-64	54 (56)	124 (58)	1.0 (Ref)	--	
≥65	43 (44)	90 (42)	0.91 (0.56-1.48)	0.707	
Gender					
Male	60 (62)	80 (38)	1.0 (Ref.)	--	1.0 (Ref.)
Female	37 (38)	134 (63)	2.72 (1.66-4.45)	0.000	2.40 (1.42-4.05)
Current frequency of driving					
Everyday or almost everyday	87 (90)	161 (75)	1.0 (Ref.)	--	1.0 (Ref.)
Occasionally	7 (7.2)	40 (19)	3.08 (1.33-7.18)	0.009	3.52 (1.39-8.91)
Seldom	3 (3.1)	13 (6.1)	2.34 (0.65-8.44)	0.193	1.73 (0.45-6.61)
Self-rated general driving ability					
Good	93 (96)	194 (91)	1.0 (Ref)	--	1.0 (Ref.)
Average	3 (3.1)	18 (8.4)	2.88 (0.83-10.01)	0.097	2.10 (0.58-7.66)
Poor	0 (0.0)	1 (0.5)	--	--	--
≥1 MVC (as a driver) in past 12 months	8 (8.3)	21 (9.8)	1.23 (0.53-2.90)	0.626	
≥1 police stops (as a driver) in past 12 months	15 (15)	44 (21)	0.87 (0.66-1.16)	0.266	
In past 12 months, asked by someone to stop driving	1 (1.0)	5 (2.3)	0.43 (0.05-3.75)	0.446	
Frequency of feeling confused, nervous or uncomfortable while driving					
Never	74 (76)	126 (59)	1.0 (Ref.)	--	1.0 (Ref.)
Rarely/Often/Sometimes	22 (23)	83 (39)	2.22 (1.28-3.84)	0.005	1.87 (1.03-3.39)
Frequency of having vision problems while driving^‡^					
Never	89 (92)	170 (79)	1.0 (Ref.)	--	1.0 (Ref.)
Rarely/Often/Sometimes	7 (7.2)	39 (18)	2.92 (1.25-6.79)	0.013	2.15 (0.88-5.26)

**Table** **2** **Legend:**

95% CI: 95% Confidence Interval; MVC: Motor Vehicle Crash.

*Odds ratio per increased year of age.

^†^Participants were asked if they “usually avoid driving” in each of the following situations, with multiple “yes” responses allowed: at night; on high speed roads; in heavy traffic; in bad weather; with others; alone; or in unfamiliar places.

^‡^Participants were asked “While stopped in a vehicle at a traffic light, how often do you have trouble reading the license plate on the care in front of you? (This means when wearing glasses or contacts, if needed)”.

Numbers may not add to 100% or total because of rounding or missing data (not reported if <5% of total).

## Discussion

In this sample of ED patients who were current drivers, over two-thirds reported self-restricting driving in at least one hazardous situation, most commonly in bad weather. Notably, neither age in years nor age group (25–64 years versus ≥65 years) was an independent predictor of self-restricted driving. To date, there have been numerous studies examining self-restricted driving patterns among older adults, with a common assumption the behavior develops in older age and older drivers self-restrict more than younger drivers. Our study suggests, however, that at least in some populations, the two groups may self-restrict more similarly than previously recognized. This may be a factor related to the medical illnesses that were associated with their ED visit, since self-restriction may be more dependent on medical conditions than age *per se*. However, older drivers more commonly avoided driving at night or on high-speed roads, and women were more likely to self-restrict driving than men. These findings add to the growing body of knowledge concerning self-restriction and provide novel comparison information from younger drivers in ED settings.

Numerous studies have documented that many older drivers adopt self-regulatory behaviors by limiting their driving in certain hazardous situations or by decreasing their total mileage or frequency of driving (Betz and Lowenstein [2010]; Braitman and McCartt [2008]; Braitman and Williams [2011]; Charlton et al. [2003]; Donorfio et al. [2008a]; Forrest et al. [1997]; Lyman et al. [2001]; Molnar et al. [2014]; Naumann et al. [2011]). In this study, we found that 1.5-49% of older drivers reported usually avoiding driving in various conditions. Possible reasons for self-restriction include compensation for particular deficits (e.g., avoiding night driving because of poor vision), a general common sense approach to traffic safety (e.g., avoiding driving in very bad weather; Charlton et al. [2006]), a change in lifestyle (Molnar et al. [2013]), or individual characteristics like gender or personality (Gwyther and Holland [2012]; Molnar et al. [2013]). In this study, we found that older drivers were more likely to self-restrict driving if they reported feeling confused, nervous or uncomfortable when driving, which is consistent with prior work suggesting that at least some older drivers are more likely to self-regulate driving when they recognize cognitive or visual deficits (Braitman and McCartt [2008]; Braitman and Williams [2011]; Lyman et al. [2001]; Stutts [1998]; West et al. [2003]). While it is clear that many older drivers do limit their driving, it unlikely that such self-regulation alone can be relied upon to ensure older driver safety, as our work supports those of others suggesting that self-regulatory practices may be more related to personality than age alone (Molnar et al. [2013]; Nichols et al. [2012]).

To date, almost all studies of self-restricted driving have sampled drivers aged 55 years and older, including a recent review of the association between personality and driving that focused on drivers aged 40 years and older (Nichols et al. [2012]). In most of these studies of older drivers, increasing age has been associated with increased self-restriction in particular conditions or as decreased mileage or frequency (Braitman and McCartt [2008]; Braitman and Williams [2011]; Charlton et al. [2006]; Donorfio et al. [2008a]; Forrest et al. [1997]; Molnar et al. [2014]; Naumann et al. [2011]). In a previous small sample in one ED, drivers aged ≥65 years were more likely than those aged 18–64 to self-restrict in all situations, although detailed analyses on age and self-restriction interactions were not performed (Betz and Fisher [2009]). In the current larger sample of drivers aged ≥25 years, age was not a significant independent predictor of self-restricted driving when examined as a continuous variable or by age group. In fact, we found that younger drivers were just as likely as older drivers to report avoiding driving in at least one hazardous situation (70% versus 68%, respectively). There were differences by age with respect to the particular situations avoided, however, with older drivers more likely than younger drivers to report avoiding driving at night (49% versus 22%) or on high speed roads (26% versus 7%). In a recent study using a national survey, Naumann et al. ([2011]) found the same trend among younger and older drivers with similar proportions for avoiding driving at night (50% for older drivers versus 23% for younger drivers), but slightly higher estimates for avoiding driving on high speed roads (29% for older drivers versus 17% for younger drivers). We found that similar proportions of older (45%) and younger (48%) drivers reported avoiding driving in bad weather, which is also similar to estimates from Naumann (54% for older drivers versus 46% for younger drivers). Unfortunately, the survey used in the Naumann study did not ask younger drivers about self-restriction under other conditions, so our findings concerning younger driver avoidance of driving in heavy traffic, unfamiliar places, with others or when alone are novel. For these situations, we found no significant differences between older and younger drivers, but additional studies with larger, community samples would be useful to examine more closely possible age-differences in self-restricted driving patterns.

We found that female gender was an independent predictor of self-restricted driving in the sample as a whole and among older drivers in stratified analysis. This is consistent with prior studies showing that older women drivers are more likely to drive less, to avoid driving in certain situations (Betz and Lowenstein [2010]; Charlton et al. [2003]; Charlton et al. [2006]; Donorfio et al. [2008a]; Gwyther and Holland [2012]; Kostyniuk and Molnar [2008]; Lyman et al. [2001]; West et al. [2003]) or to cease driving earlier than men (Carr et al. [2006]). Others have hypothesized that this gender difference may relate to lower confidence among women drivers (Donorfio et al. [2008a]; Kostyniuk and Molnar [2008]) and have noted that women may be more likely to voluntarily give up their licenses prematurely (Siren et al. [2004]). However, qualitative research has suggested that older women may have a more realistic appreciation of the fact that driving ability can change with age and may be more open to retraining courses to improve driving (Donorfio et al. [2008b]). Our study supports the findings of Naumann et al. ([2011]) that even younger women are more likely to self-restrict their driving. More studies are needed regarding gender differences and interventions to maintain and improving confidence in older adults to extend driving life expectancy.

Our sample focused on ED patients because we hypothesized that this may represent a population that typically has more comorbidities (Institute of Medicine IOM Committee on the Future of Emergency Care in the United States Health System [2007]), possibly increased risk of adverse driving outcomes, and perhaps different patterns of self-restricted driving compared to the general population. This is also a population that may not be captured through other healthcare settings, if patients lack medical insurance or choose to use ED settings as their primary site for medical care (Institute of Medicine IOM Committee on the Future of Emergency Care in the United States Health System [2007]). In addition, patients with falls or other trauma-related ED visits may be at-risk for a future MVC, or perhaps an MVC was the primary reason for the ED visit. Thus, an ED visit may be the “sentinel” event at which to identify those patients who are not restricting or may have an increased MVC risk and to educate them or refer them for interventions to reduce their crash risk (Lotfipour et al. [2014]; Stiffler and Wilber [2011]).

We found a non-significant trend towards a greater likelihood for younger drivers to report ≥1 MVC as a driver in the past 12 months (11% versus 6.8% for older drivers). This is consistent with national estimates for MVC rates per 100 licensed drivers (7% for drivers aged 25–64, 4% for drivers aged ≥65 years (U.S. Census Bureau [2012]), although the current study’s estimates are higher than national figures, but lower than estimates from a different ED study (Betz and Fisher [2009]) for both age groups. In this sample, younger drivers were more likely to report ≥1 police stop as a driver in the past 12 months (25% versus 11% for older drivers), which is consistent with findings from a prior, smaller ED sample (20% for younger versus 4.1% for older drivers; (Betz and Fisher [2009]). These findings suggest that both younger and older ED patients may be at higher risk of MVCs than the general population, which has implications for future counseling or other injury prevention efforts in ED settings.

### Limitations

Our study does have limitations, including that the findings from a sample of ED patients recruited during daytime hours at a tertiary referral hospital may not generalize to other settings or other populations. However, many of our results were similar to available national estimates. We excluded patients with acute critical illness or cognitive dysfunction, who may differ from participants in terms of baseline health or other characteristics that could influence driving patterns, but we did have high participation rates among eligible younger and older drivers. We relied on anonymous self-report without independent verification for both our predictor and outcome variables, but we chose this method for feasibility and to facilitate honest responses. We pilot-tested the survey for content and clarity but other metrics concerning question validity were not available. We cannot rule out that our screen may not have identified individuals with mild dementia where histories would possibly have been inaccurate. Certain variables such as mileage estimates or confidence ratings in various driving situations were not included on the survey because we sought to use a short instrument to increase participation. However, we acknowledge that some of these variables may be associated with self-restriction. We also did not collect information about medications received during the ED visit, the reason for the ED visit, or specific recent diagnoses or injuries, all of which might affect participant responses or driver behavior and could be interesting to examine in future studies. We recognize that participant health and ED utilization (including as related to access to other care settings) may vary between age groups, which raises the possibility of confounding. In addition, the younger and older groups were recruited during different time periods, so seasonal or other temporal factors may have influenced results. We did not assess the reliability of responses, although during acute illness answers may differ compared to when patients are at their baseline state of “wellness.” We acknowledge that, given our relatively small sample size and limited number of outcomes, our model may be at risk of overfitting and should be considered hypothesis-generating. Our method of dichotomizing variables may have overestimated the effect of cognitive problems or vision on self-restriction, as those who reported “rarely” having problems were included as positive responses. Additional work is needed to develop screening or assessment tools to identify ED drivers who may be at especially high risk of crash and to develop targeted interventions for these higher-risk groups. In addition, studies to describe the prevalence and patterns of driving self-restriction among younger and older drivers in community and other clinical settings would be useful, as would targeted research to better elucidate whether and how age affects driving self-restriction or whether observed patterns are mostly attributable to age-independent personality traits.

## Conclusion

In this sample of ED patients, we found that two-thirds of both younger and older drivers reported usually avoiding driving in at least one hazardous situation. Consistent with prior work, women were more likely to report self-restricting their driving, especially among older adults. Contrary to some prior studies, age alone was not an independent predictor of self-restricted driving, although older drivers were more likely than younger drivers to avoid driving at night and on high speed roads and to avoid a greater number of conditions. In both age groups, a greater proportion of drivers reported being involved in an MVC in the past 12 months as compared to national estimates. These findings suggest that there may be an elevated crash risk among ED patients and needs further verification with larger samples to determine whether further screening and interventions are warranted. Self-restricted driving does not appear to be a phenomenon limited to the older drivers in the ED setting, which may have implications for educational and licensing strategies to reduce crash risk in ED patients, and self-restriction may not be the optimal focus for driving safety programs or educational materials for older ED patients.

## Authors’ contributors

MEB participated in study concept and design, data acquisition, analysis and interpretation, and manuscript preparation, and she takes responsibility for the manuscript as a whole. EG participated in patient enrollment and data management. CC and DC participated in analytic design, interpretation of data, and preparation of manuscript. All authors read and approved the final manuscript. We thank Erin Rodgers and Dan Balk for their assistance with patient enrollment.

## Additional file

## Electronic supplementary material

Additional file 1: **Survey Questions.** (DOC 33 KB)

Below are the links to the authors’ original submitted files for images.

Authors’ original file for figure 1
