# Supplementary material for: Driving self-restriction and age: a study of emergency department patients
Source: Inj Epidemiol. 2014 Sep 2;1(1):18. doi: 10.1186/s40621-014-0018-z (PMC4580257; doi:10.1186/s40621-014-0018-z)
Supplement: Supplementary file 1 — Additional file 1: Survey Questions. (DOC 33 KB) [file 40621_2014_18_MOESM1_ESM.doc]

**Appendix: Survey Questions**

1. How often do you currently drive a motor vehicle?

- Every day or almost every day
- Occasionally
- Seldom

2. Would you say that in general, your driving ability is:

- Good
- Average
- Poor

3. Some people try not to drive in certain circumstances. Please tell us if you usually avoid driving at the following times (check all that apply):^1^

- At night
- When you are alone in the car
- When someone else is in the car
- When there is a lot of traffic
- On interstate highways or high speed roads
- When driving in unfamiliar places
- In bad weather (rain, snow, sleet, hail, or fog)

4. In the past **12 months**, has anyone (family members, friends, or others):

- Yes □ No Talked to you about driving safety?
- Yes □ No Restricted you from driving with passengers?
- Yes □ No Recommended you stop driving or give up your car keys?

5. In the past **12 months**, how many times have you been stopped by a police officer while driving? ^2^

Number_________

6. In the past **12 months**, how many car accidents have you been involved in (in which you were the driver, either at-fault or not)?^2^ Number_________

7. When you’re driving, how often do you feel confused, nervous, or uncomfortable?^3^

- Often
- Sometimes
- Rarely
- Never

8. While stopped in a vehicle at a traffic light, how often do you have trouble reading the license plate on the care in front of you? (This means when wearing glasses or contacts, if needed) ^4^

- Often
- Sometimes
- Rarely
- Never

*1. Second injury control and risk survey (ICARIS-2). Atlanta, GA: United States Department of Health and Human Services, Centers for Disease Control and Prevention.* [*http://www.cdc.gov/ncipc/osp/icaris2.htm*](http://www.cdc.gov/ncipc/osp/icaris2.htm)*.*

*2. Owsley C, Stalvey B, Wells J, Sloane ME. Older drivers and cataract: Driving habits and crash risk. J Gerontol A Biol Sci Med Sci. 1999;54(4):M203-11.*

*3. Betz ME, Fisher J. The trail-making test b and driver screening in the emergency department. Traffic Inj Prev. 2009;10(5):415-20.*

*4. Behavioral risk factor surveillance system survey questionnaires. Atlanta, GA: Centers for Disease Control and Prevention, National Center for Chronic Disease Prevention and Health Promotion; 2009.* [*http://www.cdc.gov/brfss/*](http://www.cdc.gov/brfss/)
