# Supplementary figures and images for: Driving self-restriction and age: a study of emergency department patients
Source: Inj Epidemiol. 2014 Sep 2;1(1):18. doi: 10.1186/s40621-014-0018-z (PMC4580257; doi:10.1186/s40621-014-0018-z)

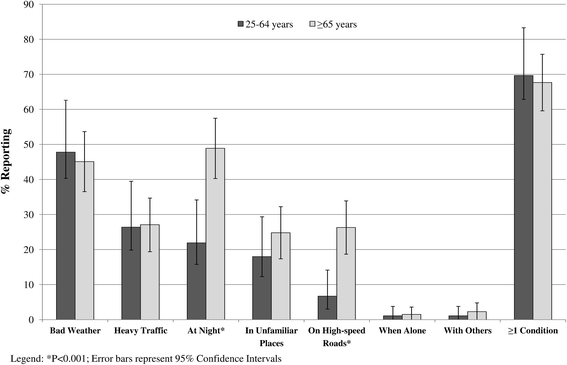

Supplement: Supplementary file 2 — Authors’ original file for figure 1 [file 40621_2014_18_MOESM2_ESM.gif]
